# Lessons on food security from the COVID-19 pandemic in Bermuda

**DOI:** 10.1371/journal.pgph.0002837

**Published:** 2024-02-12

**Authors:** Elisa Pineda, Junhui Li, Danying Li, Todd Brown, Tazeem Bhatia, Ian F. Walker, Jack Olney, Franco Sassi

**Affiliations:** 1 Department of Economics & Public Policy, Centre for Health Economics & Policy Innovation, Imperial College Business School, Imperial College London, London, United Kingdom; 2 The George Institute for Global Health, School of Public Health, Imperial College London, London, United Kingdom; 3 School of Management, Harbin Institute of Technology, Harbin, China; 4 Office for Health Improvement and Disparities, Department of Health and Social Care, United Kingdom Government, London, United Kingdom; African Population and Health Research Center, KENYA

## Abstract

Compared with other OECD countries, Bermuda ranks third globally in terms of income inequality globally. During the COVID-19 pandemic, anecdotal evidence suggested, significant fluctuations in the food demand and supply. We aimed to examine the impact of the COVID-19 pandemic on food insecurity, with a focus on the availability and affordability of various foods in Bermuda. We utilized a cross-sectional study design to investigate potential drivers of food insecurity within the local population. To gauge the level of household food insecurity we relied on the Bermuda Omnibus survey (N = 400) undertaken by Total Research Associates Ltd via telephone. To assess changes in food availability and affordability we conducted semi-structured interviews with key stakeholders who played pivotal roles in shaping food accessibility availability and affordability of food in Bermuda. These interviews were systematically analysed using the framework method. We performed analyses of food retail and import data to evaluate fluctuations in food prices and their impact on food availability and affordability. We found statistically significant associations between changes in food consumption, household income, and government aid. Food aid beneficiaries ate fewer fruits and vegetables by 50% [95% CI:17%-83%] and less fresh meat and fish by 39% [95 CI:3%-75%] compared with residents who did not receive any aid during the COVID-19 period from March 2020 to March 2021. Although we did not identify statistically significant food price increases feeding programmes played a pivotal role in preventing food insecurity during the pandemic in Bermuda. However, a lack of monitoring regarding the nutritional quality within the programmes, allowed a wide availability of foods high in sugar, salts, and fats, disproportionately affected low-income populations. In conclusion, food availability in Bermuda remained largely unaffected during the pandemic. Nevertheless, the surge in demand for feeding programs underscores underlying food security challenges in Bermuda and warrants further attention.

## Introduction

The COVID-19 pandemic in 2020 led to a 3% decline in real gross domestic product (GDP) per capita and 114 million job losses globally which may have a severe impact on food security [1]. Preceding the pandemic, approximately 9% of the global population faced food insecurity [2]. The Food and Agriculture Organization (FAO) defines food security as the consistent availability of sufficient, safe, and nutritious food, allowing individuals to meet their dietary requirements for an active and healthy life [3,4]. This definition hinges on two core dimensions: food availability, signifying the presence of ample, high-quality food through domestic production, imports, and food aid; and food affordability, referring to individuals’ access to the necessary resources to acquire nutritious foods for a healthy diet [5]. It is estimated that in 2019, around 2 billion individuals globally experienced moderate to severe food insecurity, lacking regular access to safe, nutritious, and sufficient food. This alarming trend raises concerns regarding the achievement of the second United Nations Sustainable Development Goal—Zero Hunger—by 2030. Current trajectories suggest that, by 2030, the number of individuals grappling with hunger could surpass 840 million. Preliminary assessments indicate that the COVID-19 pandemic may have added between 83 and 132 million people to the total number of undernourished individuals in 2020, contingent upon economic growth scenarios [6].

Bermuda, as revealed in the 2016 Population & Housing Census Report, has experienced persistent and expanding income inequality since 1993, affecting all forms of household income. The country’s income inequality has risen to become one of the highest globally, ranking third among all OECD countries [7]. Additionally, Bermuda has 11% of its population under the poverty line and experiencing food insecurity even before the onset of the COVID-19 pandemic [8].

A diagnostic test confirmed the arrival of the COVID-19 pandemic in the British Overseas Territory of Bermuda on March 18, 2020 [9]. After COVID-19 reached Bermuda, the government instituted a 14-day, 24-hour "Shelter in Place" order, effective from April 4th, which restricted residents to leaving their homes only for essential needs like grocery shopping, acquiring pharmaceuticals, or addressing medical emergencies. On April 14th, an extension of 14 more days for the "Shelter in Place" directive was announced [10]. Food imports were temporarily halted. Anecdotal evidence highlights substantial fluctuations in demand and supply, along with challenges related to the availability and affordability of diverse food types. Travel restrictions, restaurant closures, and organized grocery shopping schedules substantially disrupted consumer food demand. Bermuda imports approximately 90–95% of its food supply [11]. Given Bermuda’s limited domestic food production, any changes in food imports and uncertainties in global food supply chains exacerbated these fluctuations. Food security in Bermuda faces distinct challenges due to high operational and transportation costs, as well as a small-scale market with limited leverage over international suppliers. Bermuda’s small market size restricts the economy’s capacity to lower costs and foster competition [12].

The social inequalities and food insecurity potentially exacerbated during the COVID-19 pandemic are of particular concern. These challenges may significantly impact food availability and affordability for Bermuda’s population. Consequently, this study aimed to comprehensively assess the COVID-19 pandemic’s impact on the availability and affordability of diverse foods in Bermuda. We sought to investigate potential drivers of food insecurity from the retail sector’s perspective, evaluate the state of food security in Bermuda, and analyze the fluctuations in the availability and affordability of various food types during the 2020 pandemic.

## Methods

In this study, we used a cross-sectional study design and qualitative and quantitative methods to assess changes in food availability and affordability in Bermuda during the COVID-19 pandemic from March 2020 to March 2021. The qualitative component of the study involved a general population survey and a series of key stakeholder semi-structured interviews. The quantitative element involved detailed analyses of food retail and import data on a selection of food products (S1 Table).

We commissioned a set of questions to understand whether availability of different foods was affected during the pandemic from the perspective of the general Bermudian population; to ascertain whether households experienced fluctuations in household income levels during the period of the pandemic; and to understand to what extent this impacted the affordability of different types of food.

### Bermuda Omnibus survey

We conducted a cross-sectional study evaluating the level of household food security, including levels of food consumption, and the impact of the pandemic on food availability and affordability in the Bermudian population.

We relied on secondary data from the 2021 Bermuda Omnibus Survey (S1 Data) which is a syndicated multi-client survey of the public conducted on a quarterly basis by Total Research Associates Ltd [13]. The survey consisted of four-closed ended questions and 6 open-ended questions about the level of household food insecurity, levels of food consumption and impact of the pandemic on food availability and affordability. The questions were designed by our team of researchers and the methods and design of the survey, undertaken by Total Research Associates Ltd., consisted of telephone interviews with a representative sample of 400 Bermudian residents (one per household), aged 18 years and older. A sample of this size drawn from the population provided a sampling error accurate to within ± 4.9 percent in 19 out of 20 samples. The sample was stratified to represent the Bermudian population across key characteristics such as age, gender, ethnicity, household income, aid from government and parish of residence. Data generated from each question in the survey was analysed by key demographic subgroups including age, income, gender, education level, and ethnicity. All open-ended responses to questions were coded into meaningful categories or dichotomous variables for analysis (S1 Data).

Linear regression analyses were conducted to assess possible associations between household food security, the impact of the pandemic on food security, the level of food consumption, and respondent characteristics. Three regression models were developed to assess 1) the associations between household food security and respondent characteristics; 2) the associations between the impact of the COVID-19 pandemic on the food security and respondent characteristics; and 3) the associations between the level of food consumption and respondent characteristics.

Considered confounders included demographics of age, race, gender, Bermudian nationality (Bermudian or not), household income, geographical area (local parishes), and aid from the government (food or financial support). All analyses were carried out in STATA 15.

### Food price data analyses

We analysed the quantitative impact of the COVID-19 pandemic on food availability and prices in Bermuda by merging food price history and Consumer Price Index (CPI) data from the Department of Statistics and import goods data from the Customs Department in Bermuda [14,15]. The CPI calculation for Bermuda contained a basket of goods for 21 food groups. Price indices per month were derived from food products by the Departments of Statistics in Bermuda [14].

To evaluate the CPI data on food, we relied on methods from previous studies [16–18] and we used the price index for each category of food and calculated the percentage change in value during the pre-pandemic period (2018 to 2019) and during the COVID-19 pandemic period (January 2020 to February 2020). For example, the percentage change in quantity of import for the “bread” food group in July 2020 was calculated as shown in Equation (Eq 1).


$$\left(\frac{Q_{Bread,July\;2020}-\frac{Q_{Bread,July\;2018}+Q_{Bread,July\;2019}}{2}}{\frac{Q_{Bread,July\;2018}+Q_{Bread,July\;2019}}{2}}\right)*100$$ Eq 1

To assess whether changes in food prices might have resulted from fluctuations in the availability of products, we analysed import data at the tariff code level from the Customs Department. It was not feasible to match tariff codes and product categories due to different categorization systems. Therefore, we conducted a manual matching of tariff codes to food categories. Tariff codes provided only a broad definition of the products contained within them therefore we relied on food product and nutritional characteristics to link tariff codes to food product categories. To ensure accurate categorisation, our matching process involved a detailed analysis of each tariff code’s description against the characteristics of the 21 food categories and judging the likelihood that the products contained within a given tariff code would be categorised as falling within a given food category. For example, in categorising ’flour,’ we included tariff code 1101.000, which pertains to ’wheat or meslin flour’, and 1105.200 for ’flour, meal, powder, flakes, granules and pellets of potatoes’, among others, given their direct relevance to this category of flour products and their composition. However, we excluded code 1106.300, relating to ’flour, meal and powder of the dried leguminous vegetables of heading 07.13, of sago or of root of tubers of heading 07.14 or of the products of Chapter 8’, as it did not align with our defined category criteria of ‘flour’. The resulting data contained the matched import and price index data for 21 food categories. The primary task of assigning these codes to appropriate categories was carried out by a master coder. To ensure the highest level of accuracy and consistency in the categorisation process, the classifications made by the master coder was verified by a secondary reviewer. To quantitatively analyse the impact of the pandemic on food prices across the island, we then analysed the patterns and trends in price data, stratified by quantity and value, for each food category and compared monthly data during the pandemic period (January 2020 to February 2021) with those in the pre-pandemic period (2018 and 2019), following the same approach described in Eq 1.

### Key stakeholder interviews

To gain deeper insights into the shifts in food availability and affordability during the COVID-19 pandemic in Bermuda, we conducted semi-structured interviews between May and June 2021. Key stakeholders were purposefully chosen through a dual process involving targeted identification from government websites and suggestions received via email from individuals within the retail, government, and charitable sectors. This selection encompassed representatives from the food retail industry, Bermuda’s governmental authorities, significant figures in the food supply chain, and front-line feeding programs. The thematic areas explored during these interviews included food affordability, food availability, feeding programs, and the broader food supply landscape in Bermuda. Our research team crafted the interview questions, which subsequently underwent a review process by a team of seasoned surveyors. This collaborative effort ensured that the interview questions were refined to maintain their reliability and effectiveness in data collection. By design, the questions were open-ended, enabling the interviewees to shape the narrative based on their unique perspectives and expertise. The interview consisting of seven sections and a total of ten open-ended questions, the focus remained on comprehensively evaluating various facets of food security and its interconnected factors throughout the pandemic (S1 Data).

Interview transcriptions were reviewed against the recording for accuracy before being anonymised and uploaded to NVivo 12 Plus for storage, coding, text search, retrieval, and thematic analysis. Data analysis was conducted using inductive content analysis of interview transcripts. Interviews were coded line-by-line by two researchers from the research team (EP and TB) before being grouped into broader categories and further refined into emerging themes. A single researcher with experience in qualitative interviews (EP) conducted all key informant interviews. The study sampling frame was specifically designed to capture diversity in perspectives and all findings were reviewed and discussed with the research team.

### Ethics statement

Interviewees were invited via email to participate in the study during May and June 2021. They were provided with a study information sheet, invited to participate via email, and subsequently obtained informed written consent and signature through a Qualtrics online form. Interviews were conducted in English using the online teleconference platform Zoom as a COVID-19 transmission mitigation strategy. Authors had access to the interviews in which individual participants could be identified during and after data collection. All participants were over 18 years of age, and no remuneration was provided for their participation. Formal written consent was obtained from participants, and ethics approval was granted in Bermuda and internally (ICREC Reference number: 20IC6673).

## Results

### Bermuda Omnibus survey descriptive statistics

A total of 400 participants were included in the Bermuda Omnibus survey. Table 1 presents the characteristics of the Bermuda Omnibus participants.

**Table 1:** General characteristics of Bermuda Omnibus survey participants.

Respondent Characteristics	Sample (N)	%
**Governmental Aid** **		
Finance aid	67	17%
Food aid	11	3%
No aid	326	82%
**Household Income**		
Income below 75K	122	31%
Income between 75K and 150K	128	32%
Income above 150K	102	26%
Did not respond	48	12%
**Parish**		
Sandys/Sands & Southampton	69	17%
Warwick & Paget	94	24%
Pembroke & Devonshire	115	29%
Hamilton, Smith’s & St. George’s/St. David’s	115	29%
Did not respond	48	12%
**Gender**		
Female	206	51·5%
Male	194	48·5%
**Age**		
Age between 18 and 34	55	14%
Age between 35 and 54	161	40%
Age above 55	184	46%
**Bermudian**		
Yes	326	82%
No	71	18%
Did not respond	3	1%
**Ethnicity**		
Black	191	48%
White	133	33%
Mixed/Other Race	48	1%
Did not respond	28	7%
**Education**		
High school or below	93	23%
Some college or university	206	52%
Graduated or post-graduate degree	89	22%
Did not respond	12	3%

* Some responses fall into “Don’t know / no answer”, not all N = 400.

** Since the pandemic began, 20% residents reported that their household had received aid. Among them, the Government Temporary Pandemic Unemployment benefit was the most common.

### Household food security

Three-quarters (74%, n = 296) of these 400 participants had enough of desired foods whilst one-quarter of the studied population (24%, n = 94) indicated they had enough food, but not always the kinds of food they wanted through the COVID-19 pandemic in Bermuda. Furthermore, 1% (n = 4) of residents reported insufficient food access and another 1% (n = 4) expressed that they often had insufficient food access. Food security levels varied significantly based on household income levels and the receipt of food aid, with the highest levels of food insecurity observed in low-income households (S1 Text).

### COVID-19 impact on food security

While more than half of the respondents (55%, n = 163) reported that food in their households remained as available and affordable as before the pandemic, the remainder expressed the opposite. Among these residents (N = 134), three quarters (75%, n = 101) believed that higher food prices since the start of the pandemic made food less affordable to their households. Additionally, 60% of residents (n = 79) indicated that the impact of the pandemic on their household’s finances made food less affordable for their household. A similar portion of residents (57%, n = 76) indicated that that the food eaten in their household had changed since the start of the pandemic because certain foods were less available than before (S1 Text).

### Changes in food consumption

The dietary habits of residents during the COVID-19 pandemic exhibited variations across different food categories. A substantial proportion, comprising 60% of respondents for fruits and vegetables, 68% for fresh meat and fish, 51% for snacks, desserts, and candy, and 45% for prepared food, reported that their consumption remained consistent with pre-pandemic levels. However, a notable segment of residents, ranging from one-sixth to one-quarter of the population, experienced an increase in their food intake. Specifically, 23% (n = 90) of participants reported consuming more fresh fruits and vegetables, while 19% (n = 75) indicated an increased intake of prepared foods, including takeaways, fast food, and ready-to-eat meals from grocery stores. An additional 17% (n = 66) of respondents reported a heightened consumption of snacks, desserts, or candy. Conversely, fewer residents (12%, n = 46) disclosed an increase in their consumption of fresh meat or fish (S1 Text).

By contrast, about three-in-ten reported a decrease in their consumption of either prepared foods (31%, n = 124) or snacks, desserts, and candy (28%, n = 113), while about two in ten (18%, n = 72) consumed less fresh meat or fish. These changes collectively contributed to a net decrease in the consumption of these three food types across the population (S1 Text).

### Bermuda Omnibus survey regression analysis

#### Respondent characteristics and food security

During the COVID-19 pandemic, the household food security in Bermuda did not differ significantly by age, sex, ethnicity, education, and parish (S1 Text).

#### Respondent characteristics and food availability and affordability

The impacts of the pandemic on food security were disproportionate across households of different income groups. Mid-to-high income earners were more likely to have available and affordable food [19%; 95% CI: 3%, 35%] and [37%; 95% CI: 19%, 54%], respectively. However, households which depended on food aid were 34% [95% CI: 17%, 51%] less likely to have access to affordable and available food compared to before the pandemic. For respondents who reported lower food availability or affordability, food aid beneficiaries were more likely to attribute their constrained food security to higher food prices [30%; 95% CI: 9%, 50%], constrained household’s finances [35%; 95% CI: 14%, 56%] or lower availability of certain foods during the pandemic [46%; 95% CI: 22%, 71%], compared to those who had not received food aid. There were 108 respondents who said “No” to food availability and affordability. Among these 108, only 7 respondents (6.5%,) were food aid beneficiaries, and 101 respondents (93.5%) did not have the food aid (Tables 2 and 3, and S1 Text).

**Table 2:** Association of respondent characteristics on food availability and affordability during the pandemic.

Respondent characteristics	Food availability and affordability
High-income earners (above 150k) vs. low-income earners (below 75k)	37% (3%, 35%) more likely to say “yes”
Middle-income (75-150k) vs. low-income earners (below 75k)	19% (19%, 54%) more likely to say “yes”
Food aid beneficiaries (vs. those without food aids)	34% (17%, 51%) less likely to say “yes”

*Note*: 95% confidence intervals are in the parentheses.

**Table 3:** Association of being food aid beneficiaries on the reasons why they felt constrained food security during the pandemic.

Respondent characteristics	Attribution of constrained food security		
	**Higher food price**	**Constrained household’s finances**	**Lower availability of certain foods during the pandemic**
Food aid beneficiaries (vs. those without food aids)	30% (9%, 50%)	35% (14%, 56%)	46% (22%, 71%)

*Note*: 95% confidence intervals are in the parentheses.

### Respondent characteristics and food consumption changes

Significant associations between changes in food consumption and household income, as well as government aid, were observed. After controlling for confounding variables, including age, race, gender, Bermudian nationality, household income, geographical area (local parishes), and government aid (in the form of food or financial support), the following patterns emerged:

Specifically, individuals who received food aid consumed 50% less fruits and vegetables [95% CI: 17%, 83%] and 39% less fresh meat and fish [95% CI: 3%, 75%] compared to residents who did not receive any aid. Higher-income earners and those receiving financial aid were 17% [95% CI: 2%, 31%] and 15% [95% CI: 2%, 28%] less likely, respectively, to report reduced consumption of fruits and vegetables compared to others. Furthermore, of the 101 (93.5%) respondents who did not have the food aid, 43% were more likely to consume snacks, desserts, or candy than those not receiving any aid (Table 4 and S1 Text).

**Table 4:** Association of respondent characteristics on the changes in their food consumptions during the pandemic.

Respondent characteristics	Food Consumption		
	**Fruit and vegetables**	**Free meet and fish**	**Snacks, deserts, or candy**
Food aid beneficiaries (vs. those without food aids)	-50% (-83%, -17%)	-39% (-75%, -3%)	43% (4%, 81%)
High-income earners (vs. low-income earners)	17% (2%, 31%)	–	–
Financial aid beneficiaries (vs. without financial aid)	15% (2%, 28%)	–	–

*Note*: 95% confidence intervals are in the parentheses. “–”in the cells indicates statistically insignificance at the 95% confidence level.

### Import data analysis results

**Fig 1:**
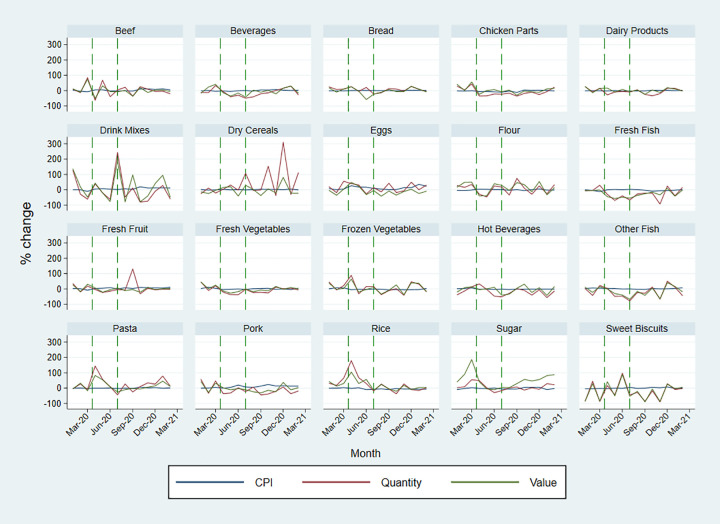
Percentage change in quantity, value of imports and the retail price level by food categories.

**Fig 2:**
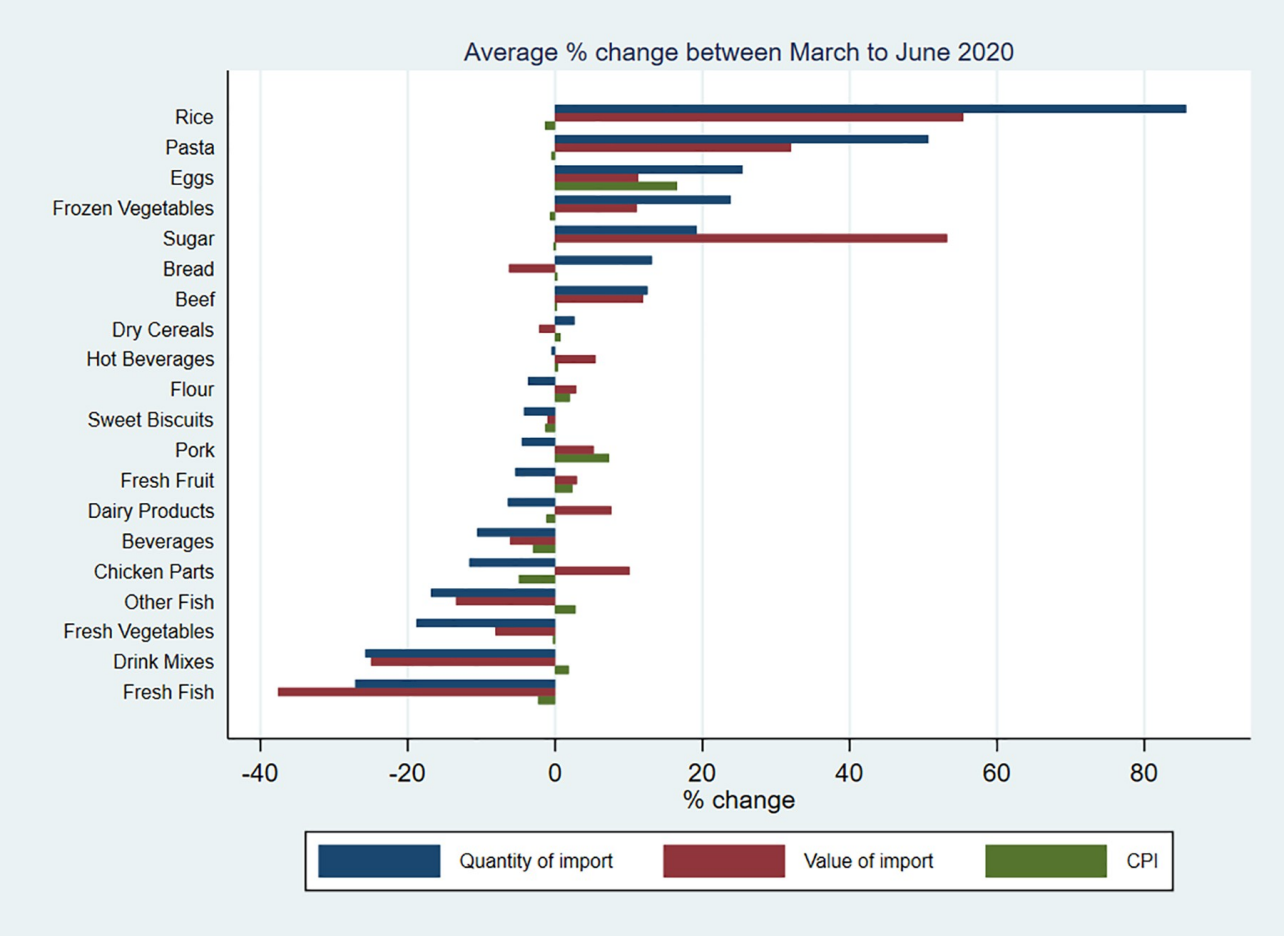
The average percentage change in quantity and value of import, and retail price level during the first lockdown by food categories.

The retail price level measured by CPI has been relatively stable during the COVID-19 pandemic and the rate of inflation has been lower than that in the US, UK, and Canada [19]. Despite increases in import price for some food categories, the changes in CPI for the same foods remained low. There were small increases in the price level of staple foods since March 2020, but then the price level has returned to its normal level or lower by February 2021. The price level for perishable foods has increased considerably and been volatile since the start of the pandemic. The price level of pork, beef and fresh fruit and vegetables were still at a high level in February 2021, being 13%, 4% 11% and 5% higher, respectively, than its previous year’s average. The price level of eggs increased by 26% in April 2020 and remained high until the end of our data observational period. Similarly, the price level of drink mixes increased since the start of the pandemic (Figs 1 and 2).

Regarding the percentage change in the number of food imports diverse trends were observed. Recalling that the first COVID-19 case in Bermuda was confirmed in March 2020, and public places including restaurants were closed from the 23^rd^ of March 2020, the quantity of import for some of the essential foods, such as rice, flour, and pasta increased considerably since. Compared with data for the same months in 2018 and 2019, the quantity of rice import increased by 66% in March, 179% in April, and 68% in May 2020, flour by 35% in March 2020, and pasta by 144% in April and 57% in May 2020, respectively. The quantity of bread import remained relatively stable over time with fluctuations being lower than 30% in general. The quantity of perishable food import (including beef, pork, chicken parts, fresh fish, dairy products, fresh fruit, and vegetables) increased significantly in March but declined in April 2020. There were also large increases in the quantity of eggs, frozen vegetables, sugar, and hot beverages import since March 2020. In contrast, the quantity of sweet biscuits, dry cereals, and drink mixes import dropped in March 2020. The fluctuations gradually declined after June 2020, possibly due to restrictions being eased and food supply chains being resumed, but there were surges for sweet biscuits in June, October, and December 2020 and February 2021, for dry cereals in July, October and December 2020 and drink mixes in July 2020, and for fresh fruit in September 2020.

Regarding the percentage change in the value of food imports, the trends of value change tracked that of quantity change; however, there were exceptions which were likely to be a result of large changes in the price level on the global trade markets. For example, in April 2020, the quantity of pork import has reduced by 36%, compared with the average quantity of pork import in 2018 and 2019, however, the value of pork import has increased by 2%. This might have indicated a large increase in the price level of pork on the import market for Bermuda in April, October, and December 2020. Similar patterns were found for chicken parts in May until August 2020, for fresh fish in November 2020, for dairy products in April and October 2020, sugar in March and after October 2020, for hot beverages in June and October 2020 and drink mixes in September 2020 and January 2021.

### Key stakeholder interview results

A total of eight key stakeholders agreed to participate in this study. Two stakeholders were from the food retail sector (R1, R2), two were from the government sector (G1, G2), and four were from front-line feeding programmes (F1, F2, F3, F4).

### Food affordability

Most participants (n = 7, 88%), believed food prices had increased during the pandemic, but only on certain food products with a minority (n = 1) believing food prices increased across the board on all products. The food products that most believed had increased in price were meat (e.g., beef, chicken, and pork), eggs and baking items (i.e., flour and yeast). Most participants agreed price increases were not immediately evident for fruits, vegetables, and ultra-processed foods (F1, F2, F3, F4, G1, G2).

From the food retailer’s perspective, the affordability of food was directly linked to the costs of the supply chain. As the cost of importing food to Bermuda increased, this cost was passed on to the consumer. Although, overall food price increases had not yet been observed by consumers, food retailers and food suppliers expected food price increases would occur in the following years.

Factors that were mentioned by key stakeholders as contributors for food price increases were associated with lumber shortages, labour shortages and higher supplier costs, which normally would not occur until January or February annually.

Due to supply disruptions for perishable food items, reduced food affordability was expected on perishable food items, especially meats. Food supplier and food retailer sector representatives (n = 3) expressed the growing challenge of complying with fresh meat (e.g., beef, chicken, and pork) availability around the globe.

Alternatively, the retail and supply sectors indicated that during the first lockdown food prices in general did not increase and, in some cases, decreased as stores switched to no-name branded products, which retailers could purchase at a lower cost. Participants did agree that at times prices would increase due to difficulties at processing plants in the US which meant supply would drop with an increase in demand leading to an increase in price mostly for meat and chicken (R1). However, the consensus between the retail and government sectors was that prices would continue to increase, and affordability would become an issue longer term particularly for low-income populations or for people whose income suddenly decreased due to unemployment (G1, G2, R1, R2) (Table 5).

**Table 5:** Themes, sub-themes, and illustrative quotes from key stakeholder interviews.

Themes and sub-themes	Illustrative quotes
**Food affordability**	
Food price increases	*“We are getting significant price increases on multiple fronts within the supply chain*. *The price of resins and plastics is at an all-time high so packaging costs are going up*. *The shortage of lumber has meant that companies are now charging for pallets that the products come on*. *So now*, *not only are we paying for the product*, *but we are also paying for what the product actually ships on…this leads to an affordability issue which will definitely be the theme of the second half of this year*.*”* (R1)
Food promotions/discounts	*“Grocery stores did offer the population a 5% discount on Wednesday’s*, *but they expanded it over the pandemic to 10% with seniors getting a discount a couple of days a week*. *So*, *they gave more*, *especially for seniors*.*”* (R1)
**Food availability**	
Food availability issues	*“The supply chain changes from the U*.*S*. *meant not being able to get certain products (e*.*g*., *meat) to the supermarkets which led to some decision to order different products*. *So*, *at times there was less on the shelves but not in any significant way where we ran completely out*.*”* (R1)
	*“Food availability was never really an issue*, *at times we couldn’t get certain brands shipped to us*, *but we were still able to get different brands*.*”* (R2)
	*“…it was the first time that I have seen such a huge community rally around local farmers and food support… as the community came together to urge the government to allow farmers to sell their produce along the roadside”* (F3).
Consumer behaviour changes	*“During the first couple of weeks of the pandemic everything and anything was being purchased … because people were not able to go to restaurants*, *they purchased more at the grocery store*.*”* (R1)
Social inequalities	“During lockdown, working-class communities had access to grocery stores only on certain days. If people don’t have transport that becomes more difficult to get to certain grocery stores. For the most stringent periods of lockdown there was not transportation running either, so people had to shop at whichever place was closest to them.” (F4)
**Feeding programmes in Bermuda**	
Government financial programmes	“…increase in the number of people requesting grocery vouchers.” (F4)
	“We don’t discriminate based on need, if someone comes and says they need food we give it to them” (F3).
	“…we paid restaurants that had closed to prepare [food] for the non-profit group because the need outstripped the capacity of the volunteers to prepare it” (F3).
Sustainability	“If the funding continues after the pandemic, then I think they will be able to continue long term.” (F2)
	*“We don’t whether the model is sustainable because I don’t know if it’s all going to be funded*.*” (F1)*
Community food growth (e.g., edible gardens)	“…big uptake of people starting the journey of growing what they could for themselves.” (G1)
**Food supply chain in Bermuda**	
Challenges and changes in the food supply chain	“…manufacturing issue and trucking issue which became an equipment shortage. So, the global supply of 20-foot or 40-foot shipping containers came to almost a standstill.” (G2)
Resilience of supply chain	*“At no point during the pandemic was Bermuda’s food security ever truly in question*. *The wide range of options and providers ensured that there was always product here*. *We responded in terms of alternate sources and by finding different shipping routes when needed to get product here*. *It was quite resilient*.*”* (G1)

Bold font denotes themes, regular font is used for sub-themes, and italics for quotes.

Stakeholder quotes: R: Retail sector G: Government sector, F: Feeding programmes.

### Food availability

Food retailer and supplier representatives expressed that Bermuda relies on imports for its food supply; therefore, food backup storage is a customary practice, which prevents food shortages. However, during the first COVID-19 lockdown period, changes that did occur on the food availability side related to the availability of certain foods and brands (Table 5). Most stakeholders agreed with this (n = 6) (with one participant declining to answer because they believed they lacked sufficient knowledge on this topic (F2).

However, food retailer stakeholders highlighted a shortage on certain foods including banana’s, flour, yeast, and meats (although meats were still accessible, just in less supply). Among the causes of this was a pest which affected supply of bananas which was independent of the pandemic (R1), whilst regarding the shortage in flour and yeast, this was suspected due to people staying home and starting to bake more as a hobby. Yeast shortage was partially overcome through retailer and hospitality joint effort and the acquirement of alternative product brands to replace and backup low availability.

Due to the closure of the hospitality sector (e.g., restaurants), food waste became a concern. Regular food supply usage was disrupted, and instances of food crop destruction were observed during the pandemic. (G1, F3, F4).

Another issue was that during the first lockdown farmers were not able to sell their produce on the side of the road. Normally, local farmers set up roadside stations to sell their food, but because of government restrictions, they were not allowed to do so (G1).

With more people working from home, changes in consumer behaviour also changed the way people accessed food in general. Getting access to food was a challenge, especially for the working-class communities during lockdown when no public transportation was running (F4) (Table 5).

### Feeding programmes in Bermuda

The feeding programmes in Bermuda provided food and resources to individuals in need. According to the Eliza DoLittle Society, there was up to a 40% increase in new registrations seeking food assistance and a 65% increase of meal provision during the first lockdown compared with the previous four months [20]. The feeding programmes, which were run by either local churches or non-profit organisations, provided meals, groceries, or food vouchers, while the government provided financial assistance if individuals lost their job or were furloughed. Other programmes also became more prevalent during the pandemic including the promotion of growing food at home.

During the interviews, every participant mentioned the feeding programmes (n = 8) and particularly an increase in their demand (F1, F2, F3, F4) (Table 5). The participants believed that either increases in food prices or a loss of income led to the increased number of people visiting the food pantry’s and participating in the feeding programmes.

Among the identified front-line feeding programmes challenges were lack of coordination between feeding programmes, effective communication networks to reach out to the community, and lack of food provision and feeding programme consumer statistics records.

The greatest concern surrounding the government financial assistance programmes came from either the homeless, those on the poverty line, and those who were part-time or did not contribute to the payroll tax as they did not qualify for assistance or the wait time to start receiving government assistance (n = 3).

In sum, during the COVID-19 pandemic in Bermuda, feeding programs exhibited significant efficacy in addressing food insecurity, as evidenced by a substantial uptick in demand. While this underscores the vital role of these programs, coverage challenges included insufficient program coordination and concerns regarding nutritional quality. However, the collaborative efforts of local churches, non-profit organizations, and the government in providing meals, groceries, and financial assistance exemplified a collective response to food insecurity. Effective and expansive, these programs were instrumental in alleviating immediate food needs. Yet, the need for continuous enhancement, particularly in terms of ensuring high nutritional quality, remains an area for further focus and investigation.

### Food supply chain in Bermuda

Functioning food supply chains are vital to Bermuda. Changes in supply chains affect food affordability and food availability. Seven of the people interviewed talked about the importance of the supply chains to Bermuda with six talking about the challenges or changes in the supply chain.

One change was in terms of brand loyalty. Certain brands had difficulties getting to Bermuda. Examples given in the interviews were brand named gluten free flours and organic flours, yeast, specific brands of chicken, certain types of crisps from the UK, cereal boxes, canned goods such as tomato paste, and smaller bags of rice (including five and 10-pound bags). Additionally, the change in shipping lines for certain foods meant they were coming from different location. A provided example was when the U.S. beef market closed, Bermuda started getting their beef from New Zealand and Brazil.

From the food supply perspective, according to interviewees, there were a couple of problems including the increase in the price of shipping and shipping container issues. The price increases came from the increase of packaging costs as mentioned by a few participants (n = 3). This included an increase in the price of lumber, resins, plastics, aluminium, etc. globally. On the other side, containers were not being filled completely due to COVID-19. Not only did this pose a food supply efficacy problem that may continue to affect the availability of food in Bermuda but may also contribute to high carbon footprint emissions and further food cost increases. However, even with the couple of challenges mentioned by the participants in this study, a common theme was the resilience of the supply chain (G1, R1, R2) (Table 5).

#### Policy recommendations

Stakeholder recommendations to address food insecurity included 1) to support, monitor and increase the efficiency of existing infrastructural programmes such as local feeding programmes and incentivising local food production through edible gardens; 2) to decrease food waste; 3) to ensure nutritional quality in the meals and foods that are provided within feeding programmes; and 4) to create community awareness of resource conservation strategies.

## Discussion

In this study, we aimed to assess the availability and affordability of food in Bermuda during the first lockdown of the COVID-19 pandemic in 2020. We conducted this study in three main phases: first, a population survey via the Omnibus Survey, second, key stakeholder interviews; and third, the analyses of food prices and imports.

Regarding the Omnibus survey, we identified that most households’ food intake and food security status had not been significantly impacted by the pandemic. Although this was true for most residents, one-third mentioned food was less available and affordable. Low-income households were more likely to experience food insecurity, consume less nutritious foods, and increase their consumption of ultra-processed foods. This may have been due to a decreased loss of income due to job losses. These results were consistent with other studies [1,21], The Caribbean outlook report by the United Nations [22], and those from the Caribbean COVID-19 Food Security and Livelihood Impact Survey [23], and the findings from our key stakeholder interviews and food price analyses.

In the semi-structured interviews, we found that interviewees agreed Bermuda was struggling more with healthy food affordability rather than overall food availability. As confirmed in the Omnibus survey, low-income groups were more likely to suffer from food insecurity and consume more unhealthy foods during the pandemic. Feeding programmes became key in the provision of food for low-income households. The feeding programmes have been a valuable resource for the prevention of food insecurity during the COVID-19 pandemic in Bermuda. A vast community effort and collaboration with the hospitality and food retail sector allowed the provision of food through pre-prepared meals and groceries to those in most need. However, focus on nutritional quality was not prioritised. Food insecurity can lead to poorer diet quality consequently increasing the risk of malnutrition [6]. Previous studies have shown that a lack of availability and affordability of healthy foods poses a higher risk for obesity and non-communicable diseases [24]. As signalled by Dimitry and Rogus [25], part of the problem may be due to the cost of healthy food and that the price of fresh fruits and vegetables is high relative to ultra-processed foods [26–30]. Therefore, the price differential between healthy and unhealthy food may help explain the link between a higher risk of obesity and NCDs, food insecurity and low-income populations. These findings coincide with those identified in a systematic review of food banks undertaken by Oldroyd et al. [31] Poor nutritional content of food programmes has been shown to be an issue in other countries [32,33]. It is essential to monitor and prioritize the provision of healthy foods in Bermuda’s feeding programs to promote healthy eating and reduce the risk of non-communicable diseases in low-income households.

The findings from the Omnibus survey and interviews were supported by the food retail and import analyses. These showed that non-processed, perishable foods such as fruits, vegetables, fish, and US suffered the most price fluctuations. A large decrease in availability was seen in these food products in April 2020 and afterwards, possibly due to global food supply chains being disrupted by the pandemic.

As observed from the key stakeholder interviews and the food price analysis, food price and availability variations may have been driven by higher demand for certain types of food during the COVID-19 pandemic. When the pandemic started, consumers stocked up on staple food items. Thus, there was a sudden and large increase in demand for these food products. Although it was observed that the supply chains for perishable food were severely affected by the pandemic, due to shortage of labour, interrupted production, and processing, as well as closures of packing plants, food prices did not statistically significantly increase in Bermuda in 2020, during the COVID-19 pandemic.

The findings observed in Bermuda are similar to other island states in the Caribbean which also rely on imports for their food supply and were also affected curing the COVID-19 pandemic. Food insecurity presents a significant challenge throughout the Caribbean region, as highlighted by a recent survey conducted by the World Food Programme. The findings revealed that 52% of households in English-speaking Caribbean countries struggle with food insecurity, although there has been some improvement compared to pre-COVID times. A key factor behind this issue is the unaffordability of food. Consequently, there are ongoing regional initiatives geared towards addressing this problem, with a goal of reducing imports by 25% to alleviate food insecurity [23].

### Strengths and limitations

This study’s limitations include using a survey conducted via telephone, potentially resulting in underrepresentation of individuals or households without a landline. Nonetheless, given Bermuda’s high phone ownership rates, the bias is limited since a minimal number of people lack phones. In January 2021, the mobile connections in Bermuda equated to 122.4% of the total population, indicating widespread phone usage [34]. While we took steps to ensure the survey’s integrity and reliability, including using a validated survey instrument and employing robust data collection methods, the inherent limitations of self-reporting must be acknowledged. However, when comparing our survey data with other sources similar findings were encountered [6,23,35]. In addition to the generalizability to the entire population of Bermuda, our study relied on a representative sample of 400 participants focused on ensuring diversity within this sample. However, certain segments of the population, such as those without access to phones, may have been excluded from our survey. Therefore, our findings should be interpreted within the context of the sampled population. This study did not specifically assess the effectiveness and coverage of feeding programs in detail as it was out of the scope of our research objectives; however, further examination of their coverage and effectiveness could be valuable in future research. In addition, due to data limitations, we could not further investigate the potential correlations between import and retail price of foods in Bermuda during the COVID-19 pandemic and were not able to make any causal inference from the descriptive results. In addition, as we were unable to gauge the extent of warehouse stock held by retailers on the island, the possible time lag between import fluctuations and retail prices changes was not considered in the analysis.

The inability to directly match imports at the product level to those sold to consumers, along with being unable to control for exogenous variables, prevents any inference of causality in these results. It is not known to what extent retailers had products in stock to replenish their shelves, which would have introduced a variable lag to the relationship between the importation of goods and any potential change in price to the consumer. A lack of sales data from retailers also prevented us from being able to analyse any trends in the volume of products being sold to consumers which may have provided additional insight into the behaviour of residents and their attitudes towards the fluctuating price of foods during the pandemic. We initially requested access to price data from all retail stores in Bermuda from the Department of Statistics. However, due to the presence of the Statistics Act 2002 [36], sharing of real-time price data from across different retail outlets is prohibited, as this would alert competitors to fluctuations in the price of specific goods and may encourage more aggressive competition. Therefore, CPI data was used, providing relative changes in price, as opposed to the absolute monetary value of products. Additionally, the matching process, whilst ensuring comprehensive coverage, also introduced inherent limitations due to subjective interpretations and the broad scope of some tariff codes, potentially affecting the granularity and specificity of our results.

The food supply in Bermuda almost entirely depends on imports. Hence, studies forecasting global food supplies beyond the pandemic may provide some insights on projecting the food security in Bermuda in the near future.

## Conclusions

While anecdotal evidence suggested imported goods became severely restricted in the early phase of the pandemic, our results indicate that the availability and affordability of various food types of foods in Bermuda were not significantly affected. While certain products may not have always been available, most of the population did not suffer from an ability to provide food for their families. However, low-income households were affected by food insecurity due to a decline in purchasing power which is likely to be linked with an increase in job losses in Bermuda which warrants further attention.

## Supporting information

S1 Data Data sources. (DOCX)

S1 Table Food import analyses. (DOCX)

S1 Text Results. (DOCX)
