# Supplementary material for: Lessons on food security from the COVID-19 pandemic in Bermuda
Source: PLOS Glob Public Health. 2024 Feb 12;4(2):e0002837. doi: 10.1371/journal.pgph.0002837 (PMC10861061; doi:10.1371/journal.pgph.0002837)
Supplement: S1 Data — (DOCX) [file pgph.0002837.s001.docx]

**Supporting Information. Lessons on food security from the COVID-19 pandemic in Bermuda**

**S1. Data Sources**

#### S1.1 Bermuda Omnibus Survey Questionnaire (1^st^ Quarter 2021)

I1. *New Question (x1 closed-ended question).* Of the four statements I am about to read you, which best describes your household’s situation with respect to food through the COVID-19 pandemic? [READ IN ORDER; CODE ONE ONLY; RE-READ AS NECESSARY]

1 Your household always had enough of the kinds of food you wanted to eat

2 Your household had enough to eat, but not always the kinds of food you wanted

3 You sometimes did not have enough food to eat ~OR~

4 You often did not have enough food to eat

**VOLUNTEERED**

98 Don’t know / no answer

I2. *New Question (x4 closed-ended question).* Next, I am going to read you four statements and I would like you to respond to each with a ‘yes’ or a ‘no’. **[RANDOMIZE ORDER; CODE ONE PER]**

1. Food in my household has been as available and affordable as it was before the pandemic.
2. Higher food prices since the start of the pandemic have made food less affordable for my household.
3. The impact of the pandemic on my household’s finances has made food less affordable for my household.
4. The food eaten in my household has changed since the start of the pandemic because certain foods are less available than before.
5. Yes
6. No

**VOLUNTEERED**

98 Don’t know/No answer

I3. *New Question (x4 closed-ended question).* For each of the following types of food, please indicate if your consumption has increased, decreased, or stayed about the same compared to what it was **before the pandemic**. **[RANDOMIZE ORDER; CODE ONE PER; REPEAT SCALE AS NECESSARY]**

1. Fresh fruit and vegetables
2. Fresh meat and fish
3. Snacks, desserts and candy
4. Prepared food, including takeaways, fast food, and ready meals from the grocery store
5. Increased
6. Decreased
7. Stayed about the same

**VOLUNTEERED**

98 Don’t know/No answer

I4a. *New Question (x1 open-ended question).* Are there any other types of food that you are now eating more of compared to before the pandemic? **[DO NOT READ; CODE ALL THAT APPLY]**

1. Canned fruits / vegetables / legumes
2. Frozen fruit / vegetables / legumes
3. Frozen meals / dinners
4. Alcohol
5. Other (Please specify:_____________)
6. None / There are no other types of food I am eating more of
7. Don’t know/No answer

I4b. *New Question (x1 open-ended question).* Are there any other types of food that you are now eating less of compared to before the pandemic? **[DO NOT READ; CODE ALL THAT APPLY]**

1. Canned fruits / vegetables / legumes
2. Frozen fruit / vegetables / legumes
3. Frozen meals / dinners
4. Alcohol
5. Other (Please specify:_____________)
6. None / There are no other types of food I am eating less of
7. Don’t know / No answer

I5. *New Question (x1 open-ended question).* **[POSE IF DECREASED IN I3A]** Why are you now eating fewer fresh fruits and vegetables? **[DO NOT READ; CODE ALL THAT APPLY]**

1. They have become more expensive
2. Trying to reduce our spending on food
3. They have become more difficult to find
4. Prefer to buy other foods instead
5. Other (Please specify:_____________)
6. Don’t know/No answer

I6. *New Question (x1 open-ended question).* **[POSE IF DECREASED IN I3B]** Why are you now eating less fresh meat and fish? **[DO NOT READ; CODE ALL THAT APPLY]**

1. They have become more expensive
2. Trying to reduce our spending on food
3. They have become more difficult to find
4. Prefer to buy other foods instead
5. Other (Please specify:_____________)
6. Don’t know/No answer

I7. *New Question (x1 open-ended question).* **[POSE IF INCREASED IN I3C]** Why are you now eating more snacks, deserts and Candy? **[DO NOT READ; CODE ALL THAT APPLY]**

1. They have become more affordable.
2. Trying to reduce our spending on food and these foods are more affordable.
3. These foods are easy to get (more available).
4. I or my household prefer these foods compared to other options.
5. Other (Please specify:_____________)
6. Don’t know/No answer

I8. *New Question (x1 open-ended question).* **[POSE IF INCREASED IN I3D]** Why are you now eating more Prepared food, including takeaways, fast food, and ready meals from the grocery store? **[DO NOT READ; CODE ALL THAT APPLY]**

1. They have become more affordable.
2. Trying to reduce our spending on food and these foods are more affordable.
3. These foods are easy to get (more available).
4. I or my household prefer these foods compared to other options.
5. Other (Please specify: _____________)
6. Don’t know/No answer

I9. *New Question (x2 closed-ended question).* Responding with a yes or a no, has your household received either of the following types of aid **that you weren’t receiving before the pandemic**? **[RANDOMIZE ORDER; CODE ONE PER]**

**[NOTE TO INTERVIEWER – DO NOT READ:** If they mention having received either of these benefits before the pandemic, please record “No”**]**

1. **Financial aid,** for example the Government Financial Assistance Programme or Government Temporary Pandemic Unemployment?
2. **Food aid,** for example food from the Eliza Doolittle Food Bank or the Hamilton Salvation Army Church?
3. Yes
4. No

**VOLUNTEERED**

98 Don’t know

99 Refused

I10. *New Question (x1 open-ended question).* **[POSE IF ‘YES’ EITHER I6 A OR B]** What kind of benefit did your household receive? **[DO NOT READ, CODE ALL THAT APPLY]**

1. Government Financial Assistance Programme
2. Government Temporary Pandemic Unemployment
3. Eliza Doolittle Food Bank
4. Hamilton Salvation Army Church
5. Hamilton Cathedral Lunchtime Feeding Programme
6. Christ Church Warwick
7. Other (Please Specify: _____________)

**VOLUNTEERED**

98 Don’t know/No answer

99 Refused

S1.2 Key stakeholders semi-structured interview schedule

Semi-structured interviews were carried out with a range of key stakeholders including representatives from the food retailers, churches and charities who provided full meals during the COVID-19 pandemic. Examples of these key stakeholders that were contacted include the Seventh Day Adventist church, the Anglican Church - Hamilton Cathedral, the Hamilton Salvation Army, and grocery store, restaurant, and supermarket representatives.

Aims

The aim of the semi-structured interviews was to understand the extent to which food was being imported onto the island and whether the balance between healthy and unhealthy foods shifted in imports and consumption.

Research question

How was food availability and affordability perceived by key stakeholders after the start of the pandemic? If there were any issues only during a specific time during the start of the pandemic, what period was this exactly?

Reference period: After the start of the COVID-10 pandemic in Bermuda.

Questionnaire 1: Food suppliers, restaurant and food and beverage retail sector

Introduction

(Re-state whilst recording that informed consent received, permission to record received)

1. Can you please provide a brief overview of your background and your business?
2. Have your customers changed their purchasing habits since the start of the pandemic? If so, how?
3. How did you adapt to these changes?
4. Were there food supply and availability problems during the reference period?
5. [if answer to Q4 highlights problems] Why do you think these problems emerged? How could these have been prevented?
6. Have supply and availability problems had an impact on your sale prices or on your ability to meet consumer demands? How did you adapt to that situation?
7. Did your business practice change in any other ways since the start of the pandemic? If so, how?
8. Has your store been involved in the delivery of feeding programmes? If yes, did you consider this type of programme was a suitable way to address food insecurity issues?

Closing

1. Finally, is there anything else you would like to tell us about how food availability and food affordability affected your business and your consumers during the reference period? Do you have any questions or any final comments?

1. Can you suggest other key stakeholders we should speak with?

*Thank you very much for your time and participating in this study.*

Questionnaire 2: Government, health, churches, and charity sector

Introduction

(Re-state whilst recording that informed consent received, permission to record received)

1. Can you please provide a brief overview of your background?
2. How did the pandemic affect food businesses and access to food for the population in Bermuda?
3. Were access to food problems caused by the reduced availability or because of reduced affordability of certain foods (e.g. higher food prices or reduced household income)?
4. If so, who do you think have been most affected?
5. Could you please tell me about the feeding programmes that were organized? What role did the feeding programmes have, who could have access to them, what were the barriers of implementation and what can you tell us about the sustainability of these programmes?
6. What went wrong and what went well with these programmes?
7. Could any changes in the food supply have facilitated and improved **food availability** during the pandemic?
8. Could any measures have been taken to improve **food affordability** during the pandemic?

Closing

1. Are there any other points that you would like to raise regarding the availability and affordability of food in Bermuda during the reference period?
2. Can you suggest other key stakeholders we should speak with?

*Thank you for your time and your availability to answer these questions.*
