# Supplementary material for: Lessons on food security from the COVID-19 pandemic in Bermuda
Source: PLOS Glob Public Health. 2024 Feb 12;4(2):e0002837. doi: 10.1371/journal.pgph.0002837 (PMC10861061; doi:10.1371/journal.pgph.0002837)
Supplement: S1 Table — (DOCX) [file pgph.0002837.s002.docx]

**Supporting Information. Lessons on food security from the COVID-19 pandemic in Bermuda**

S1 Table: Food import analyses

S2.1 Food categories and tariff codes

| Food category | Tariff code |
| --- | --- |
| Breads | 1905.100, 1905.200, 1905.400, 1905.90, 1905.901, 1905.909 |
| Sweet Biscuits | 1905.310, 1905.320 |
| Dry Cereals | 1001.110, 1001.190, 1001.910, 1001.990, 1002.100, 1002.900, 1003.100, 1003.900, 1004.100, 1004.900, 1005.100, 1005.900, 1007.100, 1007.900, 1008.100, 1008.210, 1008.290, 1008.300, 1008.400, 1008.500, 1008.600, 1008.900, 1103.110, 1103.130, 1103.190, 1103.200, 1104.120, 1104.190, 1104.220, 1104.230, 1104.290, 1104.300, 1806.901, 1901.100, 1904.100, 1904.101, 1904.109, 1904.200, 1904.300, 1904.900 |
| Rice | 1006.100, 1006.200, 1006.300, 1006.400 |
| Flour | 1101.000, 1102.200, 1102.900, 1105.100, 1105.200, 1106.100, 1106.200, 1106.300, 1901.200 |
| Pasta | 1902.110, 1902.190, 1902.200, 1902.300, 1902.400 |
| Beef | 0201.100, 0201.200, 0201.300, 0202.100, 0202.200, 0202.300 |
| Pork | 1602.410, 1602.420, 1602.490, 0203.110, 0203.120, 0203.190, 0210.110, 0210.120, 0210.190, 0203.210, 0203.220, 0203.290 |
| Chicken Parts | 0207.110, 0207.120, 0207.130, 0207.140 |
| Fresh Fish | 0302.110, 0302.130, 0302.140, 0302.190, 0302.210, 0302.220, 0302.230, 0302.240, 0302.290, 0302.310, 0302.320, 0302.330, 0302.340, 0302.350, 0302.360, 0302.390, 0302.410, 0302.420, 0302.430, 0302.440, 0302.450, 0302.460, 0302.470, 0302.490, 0302.510, 0302.520, 0302.530, 0302.540, 0302.550, 0302.560, 0302.590, 0302.710, 0302.720, 0302.730, 0302.740, 0302.790, 0302.810, 0302.820, 0302.830, 0302.840, 0302.850, 0302.890, 0302.910, 0302.920, 0302.990, 0304.310, 0304.320, 0304.330, 0304.390, 0304.410, 0304.420, 0304.430, 0304.440, 0304.450, 0304.460, 0304.470, 0304.480, 0304.490, 0304.510, 0304.520, 0304.530, 0304.540, 0304.550, 0304.560, 0304.570, 0304.590, 0307.420 |
| Other Fish | 0301.110, 0301.190, 0301.910, 0301.920, 0301.930, 0301.940, 0301.950, 0301.990, 0303.110, 0303.120, 0303.130, 0303.140, 0303.190, 0303.230, 0303.240, 0303.250, 0303.260, 0303.290, 0303.310, 0303.320, 0303.330, 0303.340, 0303.390, 0303.410, 0303.420, 0303.430, 0303.440, 0303.450, 0303.460, 0303.490, 0303.510, 0303.530, 0303.540, 0303.550, 0303.560, 0303.570, 0303.590, 0303.630, 0303.640, 0303.650, 0303.660, 0303.670, 0303.680, 0303.690, 0303.810, 0303.820, 0303.830, 0303.840, 0303.890, 0303.910, 0303.920, 0303.990, 0304.610, 0304.620, 0304.630, 0304.690, 0304.710, 0304.720, 0304.730, 0304.740, 0304.750, 0304.790, 0304.810, 0304.820, 0304.830, 0304.840, 0304.850, 0304.860, 0304.870, 0304.880, 0304.890, 0304.910, 0304.920, 0304.930, 0304.940, 0304.950, 0304.960, 0304.970, 0304.990, 0305.100, 0305.200, 0305.3100, 0305.320, 0305.390, 0305.410, 0305.420, 0305.430, 0305.440, 0305.490, 0305.510, 0305.520, 0305.530, 0305.540, 0305.590, 0305.610, 0305.620, 0305.630, 0305.640, 0305.690, 0305.710, 0305.720, 0305.790, 0307.430, 0307.490, 1604.11, 1604.12, 1604.13, 1604.14, 1604.15, 1604.16, 1604.17, 1604.18, 1604.19, 1604.2, 1604.31, 1604.32 |
| Dairy Products | 0401.100, 0401.200, 0401.400, 0401.500, 0402.100, 0402.210, 0402.290, 0402.910, 0402.990, 0403.100, 0403.900, 0404.100, 0404.900, 0405.100, 0405.200, 0405.900, 0406.100, 0406.200, 0406.300, 0406.400, 0406.900, 0409.000, 0410.000, 2105.000 |
| Eggs | 0407.110, 0407.190, 0407.210, 0407.290, 0407.900, 0408.110, 0408.190, 0408.910, 0408.990 |
| Fresh Fruit | 0801.110, 0801.120, 0801.190, 0801.210, 0801.220, 0801.310, 0801.320, 0802.110, 0802.120, 0802.210, 0802.220, 0802.310, 0802.320, 0802.410, 0802.420, 0802.510, 0802.520, 0802.610, 0802.620, 0802.700, 0802.800, 0802.900, 0803.100, 0803.900, 0804.100, 0804.200, 0804.300, 0804.400, 0804.500, 0805.100, 0805.210, 0805.220, 0805.290, 0805.400, 0805.500, 0805.900, 0806.100, 0806.200, 0807.110, 0807.190, 0807.191, 0807.192, 0807.199, 0807.200, 0808.100, 0808.300, 0808.400, 0809.100, 0809.210, 0809.290, 0809.300, 0809.400, 0810.100, 0810.200, 0810.300, 0810.400, 0810.500, 0810.600, 0810.700, 0810.900, 0814.00 |
| Fresh Vegetables | 0701.100, 0701.900, 0702.000, 0703.100, 0703.200, 0703.900, 0704.100, 0704.200, 0704.900, 0705.110, 0705.190, 0705.210, 0705.290, 0706.100, 0706.101, 0706.109, 0706.900, 0706.901, 0706.902, 0706.909, 0707.000, 0708.100, 0708.200, 0708.201, 0708.209, 0708.900, 0709.200, 0709.300, 0709.400, 0709.510, 0709.590, 0709.600, 0709.700, 0709.910, 0709.920, 0709.930, 0709.990, 0714.100, 0714.200, 0714.300, 0714.400, 0714.500, 0714.900 |
| Frozen Vegetables | 0710.100, 0710.210, 0710.220, 0710.290, 0710.300, 0710.400, 0710.800, 0710.900 |
| Sugar | 1701.120, 1701.130, 1701.140, 1701.910, 1701.990, 1702.110, 1702.190, 1702.200, 1702.300, 1702.400, 1702.500, 1702.600, 1702.900, 1703.100, 1703.900, 1704.100, 1704.900, 1704.901, 1704.902, 1704.909, 1806.10, 1806.101, 1806.109, 1806.201, 1806.311, 1806.321, 1806.902 |
| Hot Beverages | 0901.110, 0901.120, 0901.210, 0901.220, 0901.900, 0902.100, 0902.200, 0902.300, 0902.400, 0903.000 |
| Beverages | 2201.100, 2201.900, 2201.901, 2201.909, 2202.10, 2202.101, 2202.109, 2202.910, 2202.990, 2203.000, 2204.100, 2204.210, 2204.220, 2204.290, 2204.300, 2205.100, 2205.900, 2206.000, 2207.100, 2207.200, 2208.200, 2208.300, 2208.400, 2208.500, 2208.600, 2208.700, 2208.900, 9801.101, 9801.102, 9801.103, 9801.104, 9801.109 |
| Drink Mixes | 2101.110, 2101.120, 2101.200, 2101.300 |

Notes: Definitions of tariff codes can be found here: <https://www.gov.bm/sites/default/files/Bermuda-Customs-Tariff-April-2021.pdf>
