# Supplementary material for: Lessons on food security from the COVID-19 pandemic in Bermuda
Source: PLOS Glob Public Health. 2024 Feb 12;4(2):e0002837. doi: 10.1371/journal.pgph.0002837 (PMC10861061; doi:10.1371/journal.pgph.0002837)
Supplement: S1 Text — (DOCX) [file pgph.0002837.s003.docx]

**S3 Results**

**
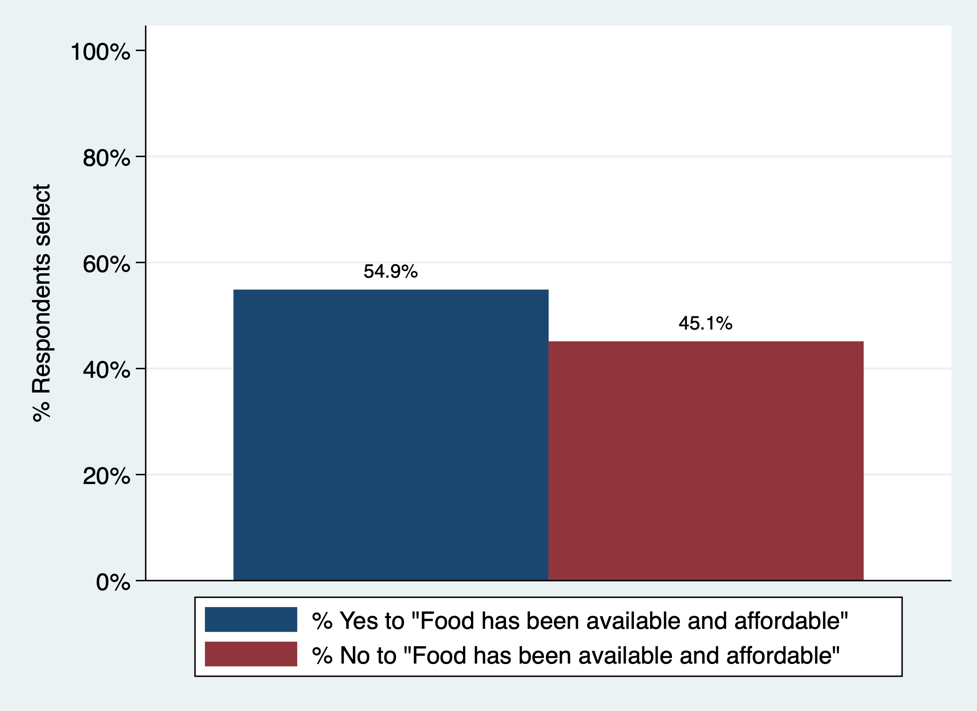
S3.1 Fig Impact of COVID 19 on Agreeing on Available and Affordable Food**


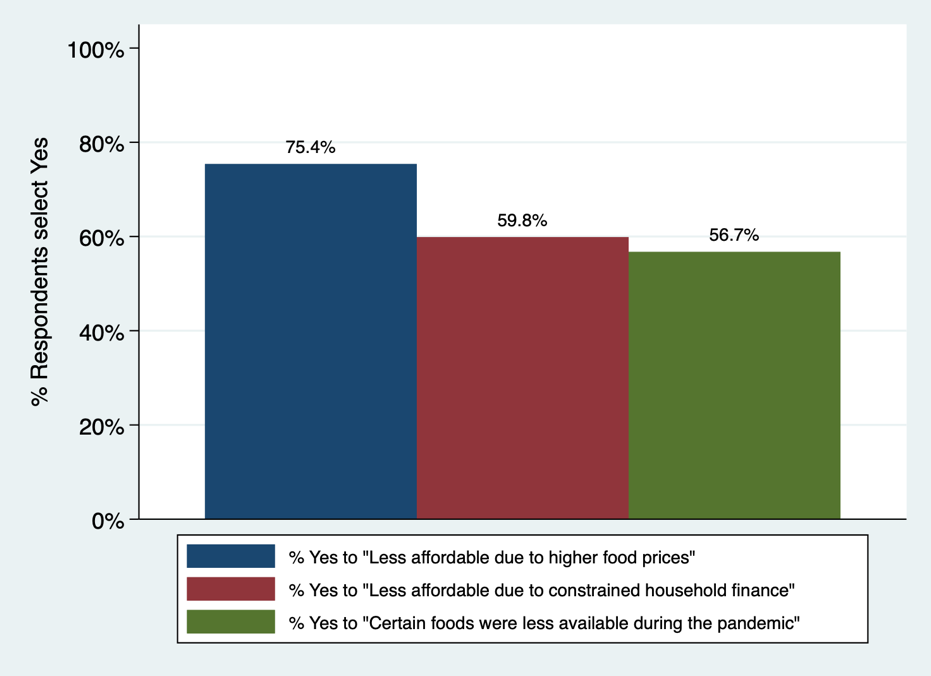
**S3.2 Fig Impact of COVID 19 on Reasons for Agreeing on Less Available and Affordable Food**

**
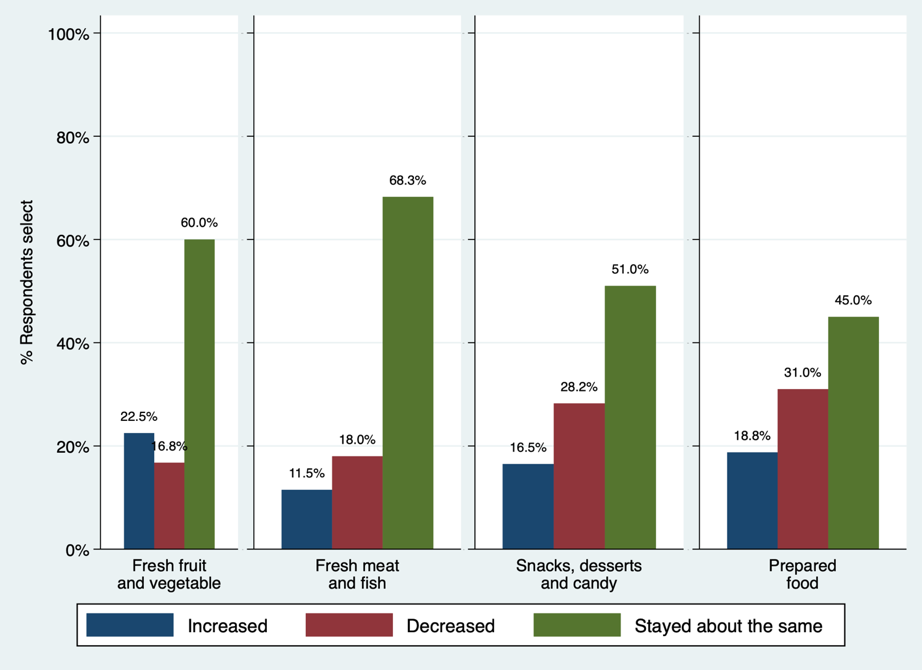
S3.3 Fig Changes in Main Food Consumptions during the COVID 19 Pandemic**

**S3.4 Reasons for food consumption changes**

Among those who reported eating fewer fresh fruits and vegetables (n=67), over four in ten (42%, n=28) mentioned that they became more difficult to find, and one-quarter mentioned they became more expensive (28%, n=19). In addition, among those who reported consuming less fresh meat and fish (n=72), one-quarter mentioned that fresh meat and fish became more expensive (25%, n=18), while more than two in ten mentioned that they became more difficult to find (22%, n=16) and a smaller portion said they were cutting down on meat (17%, n=12) (Supplementary material).

Residents consuming more snacks, desserts, and candy since the pandemic (n=66) most (32%, n=21) mentioned it was a result of being at home more/working from home, and 15% (n=10) viewed such types of food as comfort food. Further, among residents who reported eating more prepared food (n=75), most mentioned it was a result of convenience when they didn’t want to cook (28%, n=21). A smaller portion of residents mentioned that these foods were easy to get (21%, n=16), while fewer mentioned it was due to being at home more/working from home (20%, n=15). Other reasons received minor votes with respects to consuming less fruits/vegetables or meat/fish and those for eating more snacks, desserts, and candy or prepared food (Supplementary material).

**S3.5 Fig Reasons for Eating Fewer Healthy (or unhealthier eating) Food**

**S3.5.1 Fig Reasons for Eating Fewer S3.5.2 Fig Reasons for Eating Less**


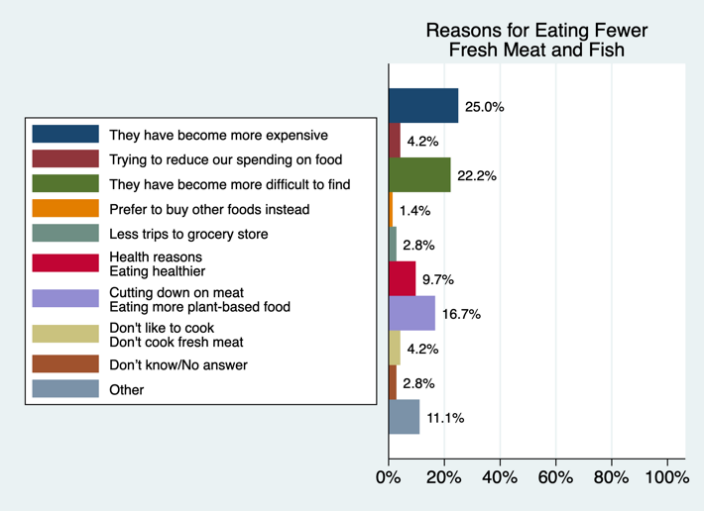

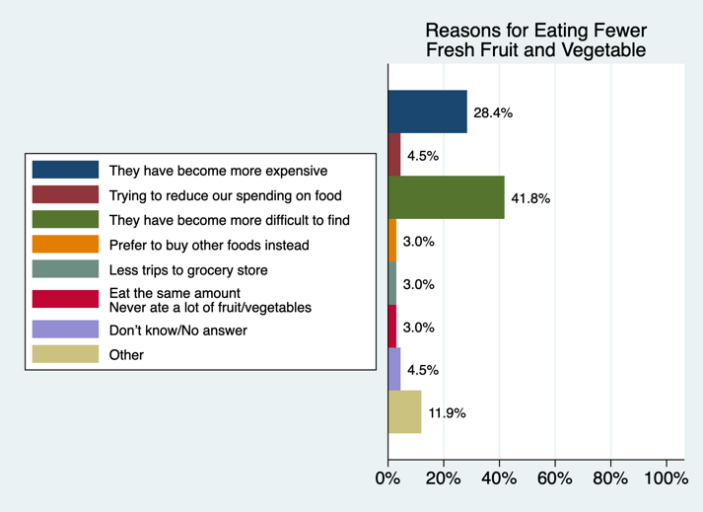
**Fresh Fruit and Vegetables Fresh Meat and Fish**

**S3.5.3 Fig Reasons for Eating More S3.5.4 Fig Reasons for Eating More**


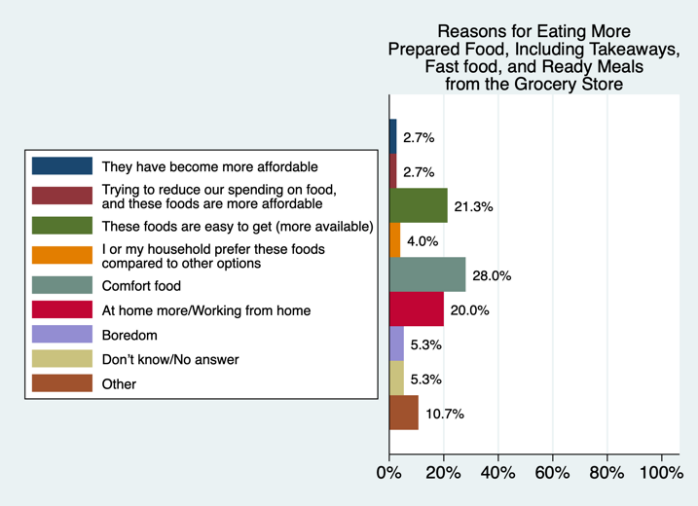

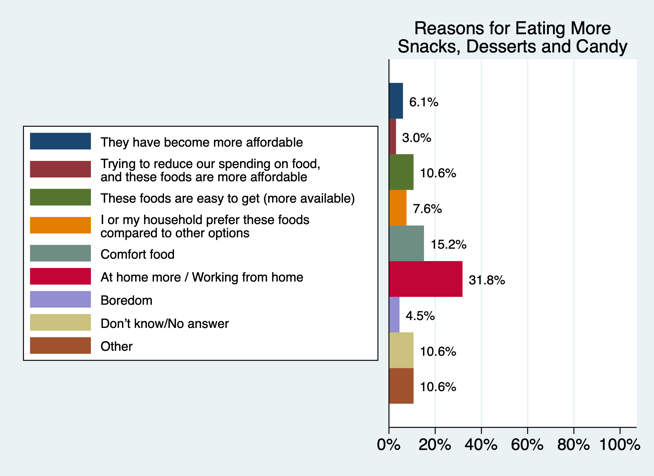
**Snacks, Deserts and Candy Prepared Food**

**S3.6 Fig Effects of Respondent Characteristics on Household Food Situation**


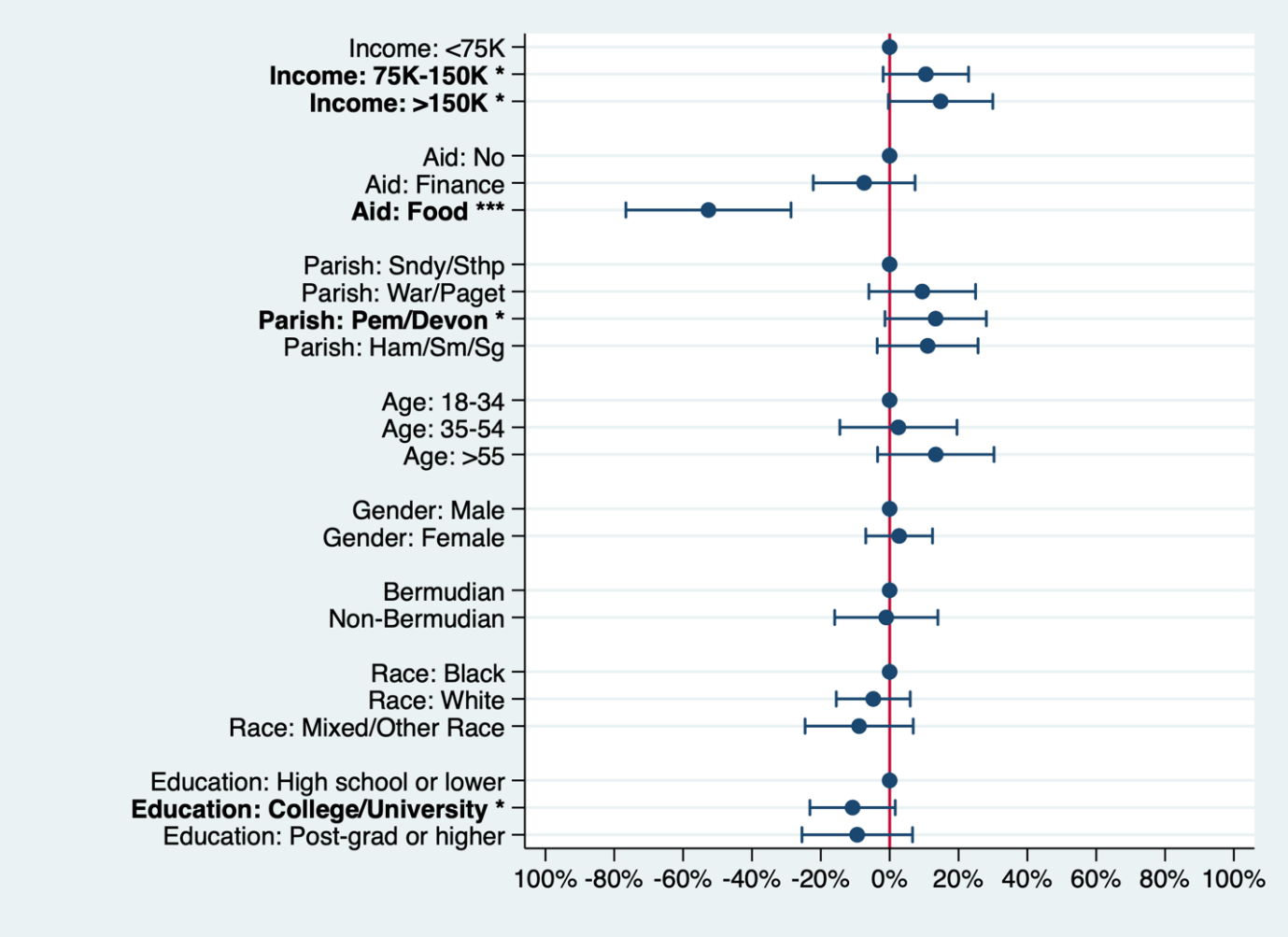


Note: 1 denotes if the household always had enough kinds of food, 0 otherwise.

*p <0.05, **p <0.01, ***p <0.001

**
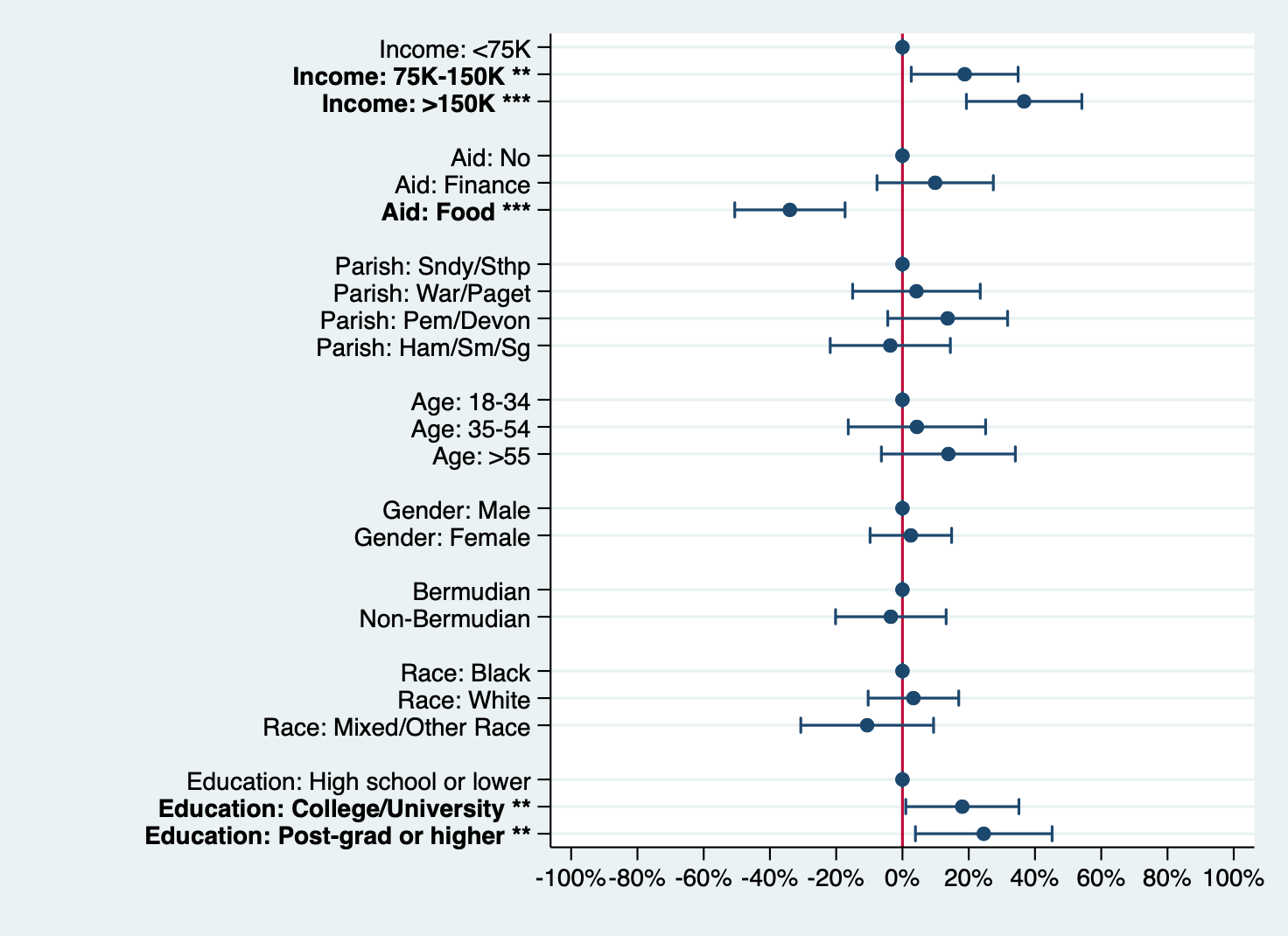
S3.7 Fig Effects of Respondent Characteristics on Agreeing on Available and Affordable Food**

Note: 1 denotes if the household always had enough kinds of food, 0 otherwise.

*p <0.05, **p <0.01, ***p <0.001

**S3.8 Fig Effects of Respondent Characteristics on Eating Fewer Healthy Food or Eating More Unhealthy Food**

**S3.8.1 Fig Effects of Respondent Characteristics S3.8.2 Fig Effects of Respondent Characteristics**

**
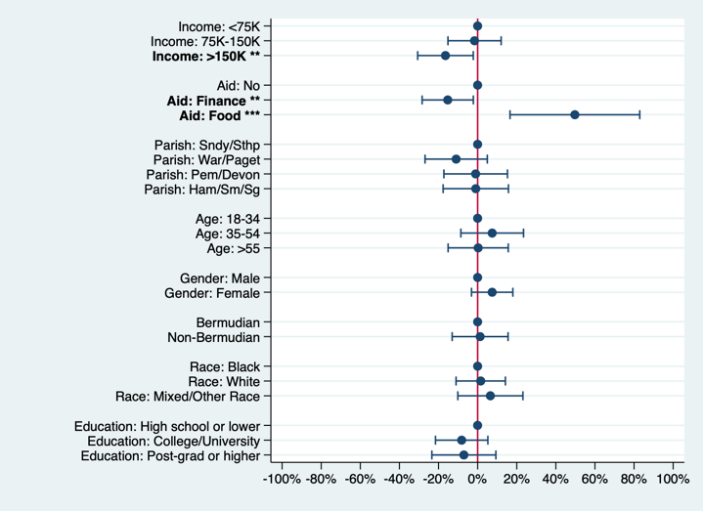

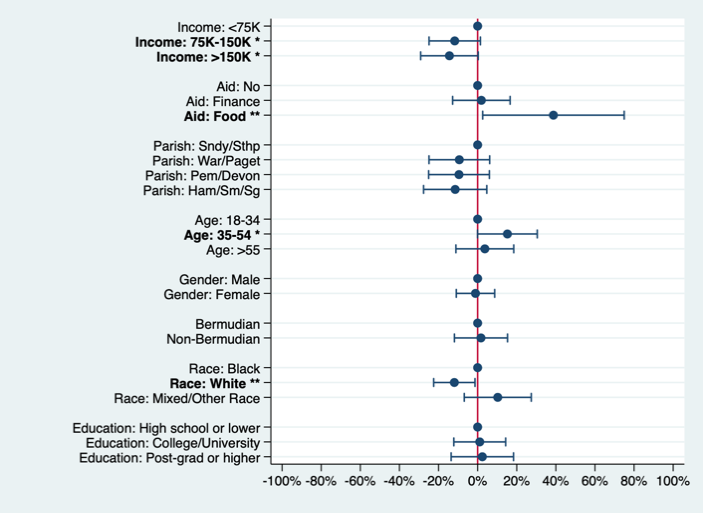
on Eating Fewer Fresh Fruit and Vegetables on Eating Less Fresh Meat and Fish**

**S3.8.3 Fig Effects of Respondent Characteristics S3.8.4 Fig Effects of Respondent Characteristics**


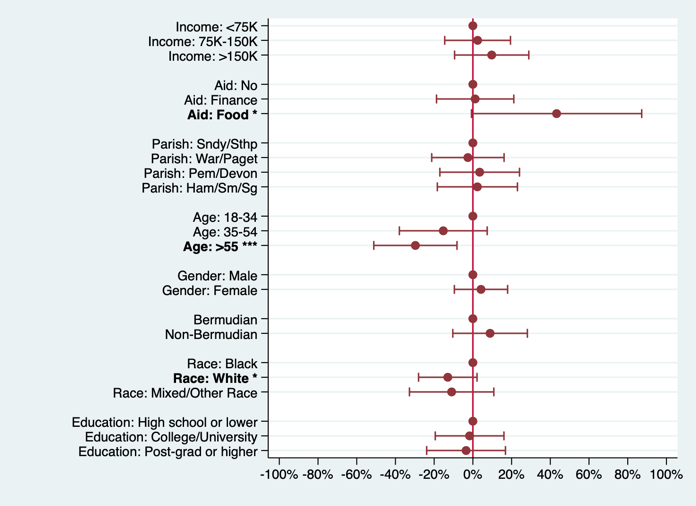

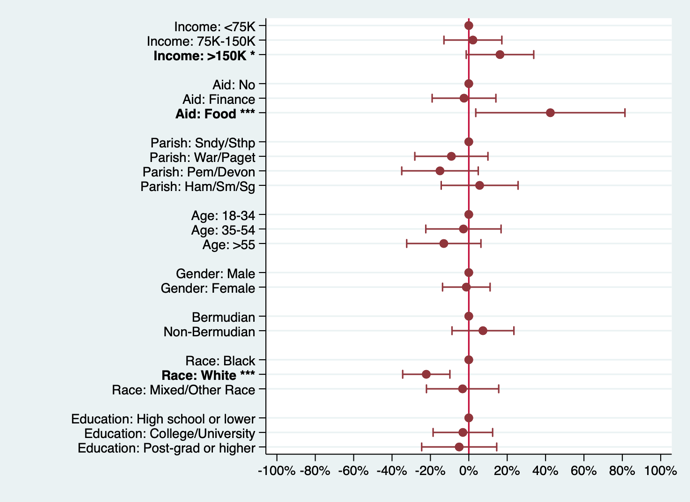
**on Eating More Snacks/Desserts/Candy on Eating More Prepared Food (D)**
